# Synthesis of phosphonate and phostone analogues of ribose-1-phosphates

**DOI:** 10.3762/bjoc.5.37

**Published:** 2009-07-27

**Authors:** Pitak Nasomjai, David O'Hagan, Alexandra M Z Slawin

**Affiliations:** 1 School of Chemistry and Centre for Biomolecular Sciences, University of St Andrews, North Haugh, St Andrews, Fife, KY16 9ST, UK

**Keywords:** 5-fluoro-5-deoxyribose-1-phosphate, fluorometabolite biosynthesis, phosphonates, phostone, ribose-1-phosphate

## Abstract

The synthesis of phosphonate analogues of ribose-1-phosphate and 5-fluoro-5-deoxyribose-1-phosphate is described. Preparations of both the α- and β-phosphonate anomers are reported for the ribose and 5-fluoro-5-deoxyribose series and a synthesis of the corresponding cyclic phostones of each α-ribose is also reported. These compounds have been prepared as tools to probe the details of fluorometabolism in *S. cattleya.*

## Introduction

**Scheme 1:**
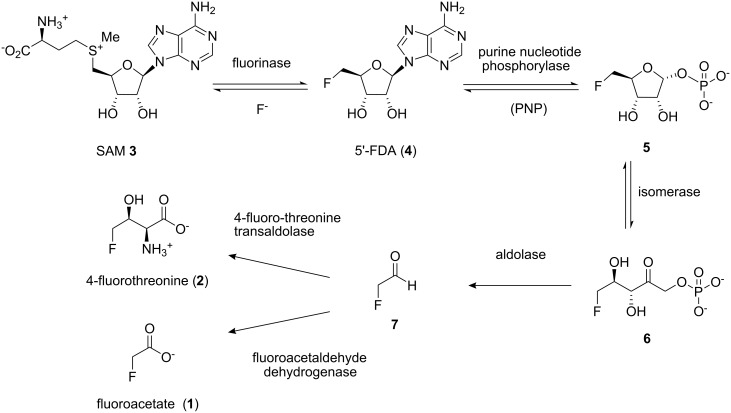
Biosynthetic pathway from fluoride ion to fluoroacetate **1** and 4-fluorothreonine **2** in *S. cattleya* [2].

Fluoroacetate (**1**) and 4-fluorothreonine (**2**) are unusual secondary metabolites in that they contain a fluorine atom. They are elaborated by the bacterium *Streptomyces cattleya* as part of its defense strategy since 4-fluorothreonine (**2**) has antibiotic activity and fluoroacetate (**1**) is a toxin [1]. The biosynthetic pathway from fluoride ion to these fluorometabolites has largely been elucidated and is summarised in Scheme 1 [2].

**Figure 1:**
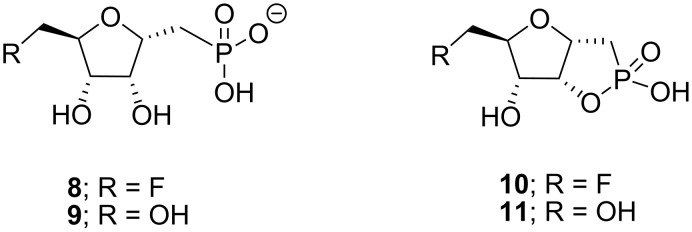
Synthesized phosphonates **8** and **9** and cyclic phostones **10** and **11**.

The first committed step in the biosynthesis involves the fluorinase, an enzyme that catalyses nucleophilic attack of fluoride ion on SAM (**3**) to generate 5′-FDA (**4**). The initial fluorinated product 5′-FDA (**4**) is then depurinated by a purine nucleotide phosphorylase (PNP) to give 5-fluoro-5-deoxyribose-1-phosphate (**5**). This phosphate intermediate becomes the substrate for a ring opening isomerisation reaction converting 5-fluoro-5-deoxyribose-1-phosphate (**5**) to 5-fluoro-5-deoxyribulose-1-phosphate (**6**). Further processing to fluoroacetaldehyde (**7**) and then conversion to fluoroacetate (**1**) and 4-fluorothreonine (**2**) complete the pathway. The focus of this research involved generating a potential inhibitor of either the PNP or the subsequent isomerase enzymes as a tool, to try to accumulate biosynthesis intermediates in cell free extract studies [3]. We have already demonstrated that the *S. cattleya* PNP can depurinate both 5′-FDA and adenosine, and thus the presence or absence of fluorine at the C-5′-position is not a requisite for catalysis. The next enzymatic step involves a ring opening isomerisation of the ribose-1-phosphate **5**. In this paper we report the synthesis of phosphonate analogues **8** and **9** of ribose-1-phosphate **5** where the linking phosphate oxygen has been replaced by a methylene group and for **9** the fluorine has been replaced by OH (see Figure 1). These phosphonates were considered as candidate inhibitors for either the PNP or the isomerase enzyme, with potential utility as tools to interrogate the biosynthesis system, and as inert candidate substrate analogues for enzyme co-crystallisation studies. We also report the synthesis of the cyclic phostone analogues **10** and **11** (Figure 1), which were viewed also as potential substrate analogues for co-crystallisation studies, particularly with the isomerase enzyme which converts **5** to **6** during the biosynthesis.

## Results and Discussion

**Scheme 2:**
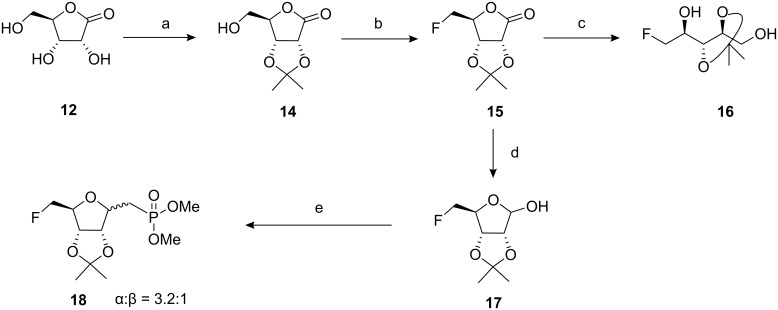
Synthetic route A. Reagents and conditions: a) acetone, concd H_2_SO_4_ (cat.), 6 h, 86%; b) Deoxo-fluor™, DCM, 40 °C, 30 min, 84%; c) NaBH_4_, ethanol, 0 °C, 2 h, 65%; d) DIBAL-H, toluene, 40 °C, 64%; e) CH_2_[P(O)(OMe)_2_]_2_, DCM, 50% aq NaOH, 60%.

The target phosphonates **8** and **9** have been prepared by routes A and B as shown in Scheme 1 and Scheme 2 respectively. The routes start either from D-ribonic-γ-lactone (**12**) or D-ribose (**13**). For each route the key synthetic steps are the same involving installation of the dimethyl phosphonate moiety on a 2,3-O-isopropylidene ribofuranoside *via* a Wadsworth-Emmons reaction [4]. Route A (Scheme 2) started with acetonide protection of D-ribonic-γ-lactone (**12**) to generate 2,3-isopropylidene **14** [5]. Fluorination of the free 5-hydroxyl group with Deoxo-fluor™ [6] gave 5-deoxy-5-fluoro-2,3-O-isopropylidene-D-ribonic-γ-lactone (**15**) in good yield and then subsequent reduction of lactone **15** with NaBH_4_ resulted in the fully reduced diol **16**. Diol **16** has been previously synthesised [7], however this route appears to offer a more efficient preparation. In our case, even with limiting NaBH_4_, mixtures of diol **16** and 5-deoxy-5-fluoro-2,3-ribofuranoside **17** were obtained, however treatment with DIBAL-H at 40 °C [8] gave a clean reaction and 5-fluororibofuranoside **17** was recovered in moderate yield. Finally a Wadsworth-Emmons reaction [4] of **17** using tetramethyl methylenediphosphonate in DCM/aq. NaOH gave a smooth conversion to 5-fluorophosphonate **18** which had an α:β ratio of 3.2:1. (The major α-anomer was assigned for **18** based on the subsequent NMR assignments of **23a** and **b**, see below.) These diastereoisomers were inseparable by conventional chromatography.

**Scheme 3:**
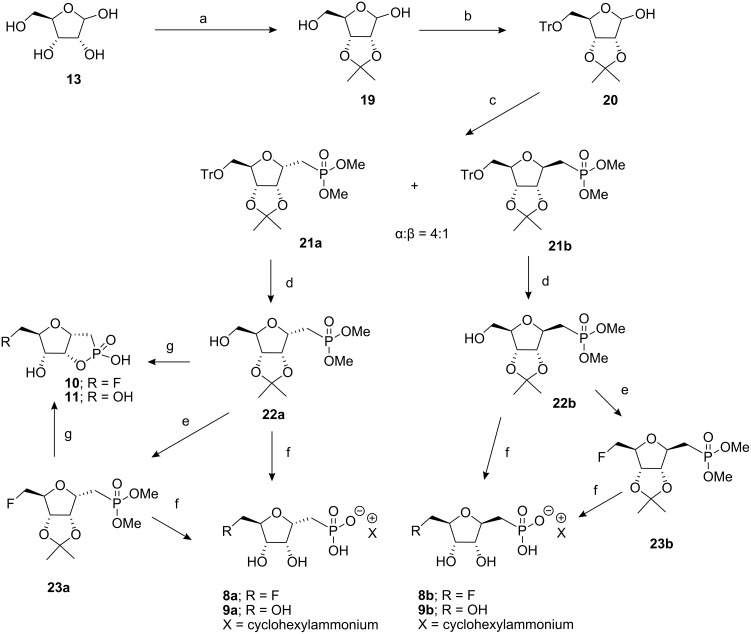
Synthetic route B. Reagents and conditions: a) acetone, concd H_2_SO_4_ (cat.), 4 h, 90%; b) TrCl, pyridine, DMAP, rt, 16 h, 94%; c) CH_2_[P(O)(OMe)_2_]_2_, 50% aq NaOH; DCM, 64%; d) anhyd ZnCl_2_, DCM, rt, 2 h, 78–81%; e) TsF, TBAF, THF, reflux, 16 h, 83–92%; f) i) TMSBr, DCM, rt; ii) TFA:H_2_O (1:1), iii) cyclohexylamine, pH ~11, 84–87%; g) i) TMSBr, DCM, rt; ii) TFA:H_2_O (1:1), iii) Ac_2_O, pyridine, 16 h, 40–47%.

**Figure 2:**
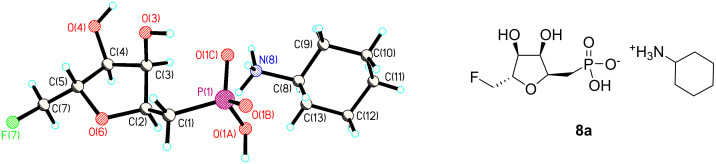
X-Ray crystal structure of cyclohexylammonium salt **8a**.

Due to the inability to separate α−**18** from β−**18**, route B (Scheme 3) was explored as an alternative. Following the protocol demonstrated by Meyer et al. [4], 5-*O*-trityl-phosphonates **21a** and **21b** were obtained as individual epimers after chromatographic separation of the initial product mixture (α:β of 4:1). Detritylation of **21a**, **b** was accomplished with ZnCl_2_ [9] to generate the 5-hydroxyphosphonates **22a** and **22b** in a good yield. The free alcohol of both epimers was then fluorinated at the 5-position using tosylfluoride and TBAF in refluxing THF [10–11]. Sequential deprotection of **23a** and **23b** with TMSBr and then TFA yielded the free phosphonic acids which were neutralised with cyclohexylamine [12] to generate the non-hygroscopic salts **8a**, **b** and **9a**, **b** which could be recrystallised from MeOH/acetone. X-Ray structure analysis of a suitable crystal of the fluoromethyl phosphonate **8a** was obtained (Figure 2) which confirmed its structure and stereochemical relationship to intermediate ribose-1-phosphate metabolite **5**. Clearly, phosphonate **8a** formed a 1:1 salt with the amine. Additionally, **23a** and **22a** were treated in separate reactions with acetic anhydride in dry pyridine [13] to generate the cyclic phostones **10** and **11** respectively. In each case this conversion could be conveniently followed by a change in the ^31^P-NMR resonance of 27 ppm (phosphonate) to 44 ppm, characteristic of a cyclic phostone. Purification by chromatography allowed the recovery of the phostones **10** and **11** in moderate yield.

Incubation of the phosphonate salts **8a** and **9a** with the *S. cattleya* PNP did not result in an inhibitory effect, and clearly the phosphonates are not good substrate analogues of the phosphates with this enzyme. We are currently exploring phosphonates **8a** and **9a** as inhibitors of the isomerase, the next enzyme on the pathway, and in order to probe the molecular mechanism of this enzyme, these phosphonates and the phostones **10** and **11** are being used in co-crystallisation trials with each of the over-expressed enzymes (PNP and isomerase).

## Experimental

Nuclear magnetic resonance (NMR) spectra were obtained using Bruker Advance 300 and Bruker Advance II 400. All chemical shifts (δ) are reported in parts per million (ppm) and coupling constants (*J*) are given in Hertz (Hz). Melting points were determined in Pyrex capillaries using a Gallenkamp Griffin MPA350.BM2.5 melting point apparatus. Infra red (IR) spectra were recorded on Nicolet Avatar 360 FT-IR. High resolution mass spectrometry (HRMS) was carried out using a Micromass LCT (Manchester, UK) mass spectrometer with electrospray ionization (ESI) operating in positive and negative modes. Optical rotations were measured on Perkin-Elmer model 341 polarimeter.

**5-Deoxy-5-fluoro-2,3-*O*-isopropylidene-D-ribono-1,4-lactone (15).** Deoxo-Fluor™ (3.06 cm^3^, 16.58 mmol) was slowly added to a stirred solution of 2,3-*O*-isopropylidene-D-ribono-1,4-lactone 14 [5] (1.04 g, 5.53 mmol) in DCM (10 cm^3^) at ambient temperature. After stirring at 40 °C for 30 min, the reaction mixture was cooled down to ambient temperature and silica gel (0.5 g) was carefully added. After removal of solvent, silica gel was applied to a silica gel column eluting with EtOAc:hexane (1:3) to afford **15** as a white solid (0.88 g, 84%); mp: 60–62 °C; [α]^20^_D_: −82.8° (*c* 5.0 × 10^−3^, CHCl_3_); ^1^H NMR (400 MHz; CDCl_3_): δ = 1.39 (s, 3H, CH_3_), 1.48 (s, 3H, CH_3_), 4.67 (ddd, 1H, ^3^*J*_HH_ = 1.9, ^2^*J*_HH_ = 11.0, ^2^*J*_HF_ = 46.1, H-5), 4.72 (dddd, 1H, ^3^*J*_HH_ = 1.7, ^3^*J*_HH_ = 1.7, ^3^*J*_HH_ = 1.7, ^3^*J*_HF_ = 34.57, H-4), 4.74 (ddd, 1H, ^3^*J*_HH_ = 1.8, ^2^*J*_HH_ = 11.0, ^2^*J*_HF_ = 48.1, H-5), 4.81 (dd, 1H, ^3^*J*_HH_ = 3.3, ^3^*J*_HH_ = 5.6, H-3), 4.85 (d, 1H, ^3^*J*_HH_ = 5.7, H-2). ^13^C NMR (100 MHz; CDCl_3_): δ = 25.5 (CH_3_), 26.8 (CH_3_), 75.1 (d, ^4^*J*_CF_ = 3.9, C-2), 77.2 (d, ^3^*J*_CF_ = 5.1, C-3), 80.3 (d, ^2^*J*_CF_ = 19.2, C-4), 82.2 (d, ^1^*J*_CF_ = 171.3, C-5), 113.9 (C(CH_3_)_2_), 173.6 (C-1). ^19^F NMR (282 MHz, CDCl_3_): δ = −236.0 (ddd, ^3^*J*_HF_ = 3.3, ^2^*J*_HF_ = 34.8, ^2^*J*_HF_ = 46.3). ν_max_ (KBr): 3563, 2993, 1804, 1455, 1384, 1272, 1054, 849, 610, 513. HRMS (ES+): Calcd. for C_8_H_11_FO_4_Na [M+Na]^+^: 213.0539, found: 213.0537.

**5-Deoxy-5-fluoro-2,3-*O*-isopropylidene-D-ribofuranose (17).** A solution of DIBAL-H (1 M in hexane, 2.20 ml, 2.18 mmol) was carefully added to a solution of 5-deoxy-5-fluoro-2,3-*O*-isopropylidene-D-ribono-1,4-lactone (**15**) (0.42 g, 2.18 mmol) in dry toluene (10 ml) at 40 °C and the reaction was monitored by ^19^F NMR. After completion, MeOH (2 ml) was added. The slurry gel was broken by adding sat. aq. solution of sodium potassium tartrate (1 ml). After extracted into ether (3 × 10 ml), the combined organic phases were washed with brine and dried over MgSO_4_. After removal of solvent under reduced pressure the residue was purified over silica gel eluting with 60% EtOAc/hexane to afford **17** as colourless oil (0.26 g, α/β = 7.6/1, 63.6%) and **16** (25.4 mg, 6.0%). **α-anomer**: ^1^H NMR (300 MHz; CDCl_3_): δ = 1.27 (s, CH_3_), 1.43 (s, CH_3_), 3.27 (t, ^3^*J*_HH_ = 5.17, -OH), 4.31–4.41 (m, 2H, H-4, H-5, (overlap with β, H-4, H-5)), 4.44–4.61 (m, 2H, H-3, H-5 (overlap with β, H-3, H-5)), 4.71 (d, ^3^*J*_HH_ = 5.9, H-2), 5.38 (dd, 1H, ^3^*J*_HH_ = 2.5, ^3^*J*_HH_ = 5.3, H-1). ^13^C NMR (75 MHz; CDCl_3_): δ = 24.9 (CH_3_), 26.5 (CH_3_), 81.2 (d, ^3^*J*_CF_ = 5.5, C-4), 82.2 (d, ^1^*J* = 171.3, C-5), 84.8 (C-3 or C-2), 86.1 (C-2 or C-3), 103.3 (C-1), 112.7 (C(CH_3_)_2_). ^19^F NMR (282 MHz, CDCl_3_): δ = −230.8 (dddd, ^4^*J*_HF_ = 3.3, ^3^*J*_HF_ = 37.5, ^2^*J*_HF_ = 47.3, ^2^*J*_HF_ = 47.3, 1F). **β-anomer**: ^1^H NMR (300 MHz; CDCl_3_): δ = 1.50 (s, CH_3_), 1.33 (s, CH_3_), 3.93 (d, ^3^*J*_HH_ = 10.9, -OH), 4.31–4.41 (m, 2H, H-4, H-5, (overlap with α, H-4, H-5)), 4.44–4.61 (m, 2H, H-3, H-5 (overlap with α, H-3, H-5)), 4.74 (ddd, 1H, ^3^*J*_HH_ = 0.8, ^3^*J*_HH_ = 1.5, ^3^*J*_HH_ = 6.4, H-4), 5.33 (ddd, 1H, ^3^*J*_HH_ = 3.7, ^3^*J*_HH_ = 3.7, ^3^*J*_HH_ = 10.9, H-1). ^13^C NMR (75 MHz; CDCl_3_): δ = 24.7 (CH_3_), 26.1 (CH_3_), 79.5 (C-2), 79.6 (d, ^2^*J*_CF_ = 17.8, C-4), 80.7 (d, ^3^*J*_CF_ = 6.7, C-3), 85.1 (d, ^1^*J* = 170.5, C-5), 97.7 (C-1), 114.2 (C(CH_3_)_2_). ^19^F NMR (282 MHz, CDCl_3_): δ = −225.4 (dddd, ^4^*J*_HF_ = 3.2, ^3^*J*_HF_ = 23.1, ^2^*J*_HF_ = 46.5, ^2^*J*_HF_ = 46.5, 1F). ν_max_ (neat): 3426, 2944, 1376, 1211, 1072, 868, 504. HRMS (ES+): Calcd. for C_8_H_13_FO_4_Na [M+Na]^+^: 215.0696, found: 215.0691.

**D-allo,altro-2,5-Anhydro-1-deoxy-1-(dimethoxyphosphinyl)-6-fluoro-3,4-*O*-isopropylidenehexitol (18).** 50% aq Solution NaOH (3 ml) was slowly added to a vigorously stirring mixture of 5-deoxy-5-fluoro-2,3-*O*-isopropylidene-D-ribofuranose **17** (0.125 g, 0.63 mmol) and tetramethyl methylenediphosphonate (0.16 g, 0.69 mmol) in DCM (3 ml) at ambient temperature. After 18 h, the reaction mixture was diluted with DCM (10 ml) and the aq phase was extracted with DCM (2 × 10 ml). The combined organic phases were dried over MgSO_4_, and filtered. After removal of solvent, the residue was purified over silica gel eluting with 5% MeOH:DCM to afford a mixture of epimers **18** as colourless oil (0.11 g, α/β = 3.2/1, 58.5%).^1^H NMR (400 MHz; CDCl_3_): δ = 1.19 (C(CH_3_)_2_), 1.33 (C(CH_3_)_2_), 1.95–2.20 (m, 2H, H-1), 3.58–3.63 (m, POCH_3_), 3.95–4.69 (m, 6H). ^13^C NMR (162 MHz; CDCl_3_): δ = 30.3–31.1 (m, P_α_), 31.7–32.4 (m, P_β_). ^19^F NMR (282 MHz, CDCl_3_): δ = −231.1 (ddd, ^3^*J* = 28.90, ^2^*J* = 47.32, ^2^*J* = 47.32, 1F_α_), −229.3 (dddd, ^4^*J* = 2.26, ^3^*J* = 32.94, ^2^*J* = 47.29, ^2^*J* = 47.29, 1F_β_). ν_max_ (neat): 3466, 2955, 1644, 1459, 1374, 1213, 1023, 822, 537. HRMS (ES+): Calcd. for C_11_H_20_O_4_FPNa [M+Na]^+^: 321.0879, found: 321.0874.

**D-altro-2,5-Anhydro-1-deoxy-1-(dimethoxyphosphinyl)-6-fluoro-3,4-*O*-isopropylidenehexitol (23a).** A solution of TBAF (1 M in THF, 2.16 ml, 2.16 mmol) was added to a mixture of **22a** (0.16 g, 0.54 mmol) and TsF (0.28g, 1.62 mmol) in THF (15 ml) and then the reaction mixture was brought to reflux. After refluxing for 18 h, the reaction mixture was cooled down to ambient temperature and then filtered through a shot pad of silica gel washing with EtOAc. After removal of solvent the residue was purified over silica gel eluting with MeOH:DCM (1:20) to afford **23a** as colourless oil (0.148 g, 92.3%). [α]_D_^20^: −16.44° (*c* 2.25 × 10^−3^, CHCl_3_). ^1^H NMR (300 MHz; CDCl_3_): δ = 1.28 (s, 3H, C(CH_3_)_2_). 1.42 (s, 3H, C(CH_3_)_2_), 2.13 (ddd, 1H, ^3^*J*_HH_ = 6.7, ^2^*J*_HH_ = 15.4, ^2^*J*_HP_ = 18.1, H-1), 2.23 (ddd, 1H, ^3^*J*_HH_ = 6.6, ^2^*J*_HH_ = 15.4, ^2^*J*_HP_ = 18.4, H-1), 3.68 (d, 3H, ^3^*J*_HH_ = 3.9, POCH_3_), 3.70 (d, 3H, ^3^*J*_HH_ = 3.9, POCH_3_), 4.12 (ddd, 1H, ^3^*J*_HH_ = 2.9, ^3^*J*_HH_ = 2.9, ^3^*J*_HF_ = 33.1, H-5), 4.26 (dddd, 1H, ^3^*J*_HH_ = 3.2, ^3^*J*_HH_ = 6.6, ^3^*J*_HH_ = 6.6, ^3^*J*_HP_ = 10.3, H-2), 4.44 (ddd, 1H, ^3^*J*_HH_ = 3.4, ^2^*J*_HH_ = 10.2, ^2^*J*_HF_ = 46.8, H-5), 4.48 (ddd, 1H, ^3^*J*_HH_ = 3.2, ^2^*J*_HH_ = 10.2, ^2^*J*_HF_ = 47.6, H-5), 4.65 (dd, ^3^*J*_HH_ = 3.9, ^3^*J*_HH_ = 6.0, 1H, H-3), 4.77 (d, ^3^*J*_HH_ = 6.0, 1H, H-4). ^13^C NMR (75 MHz; CDCl_3_): δ = 24.9 (CH_3_), 25.5 (d, ^2^*J*_PC_ = 141.9, C-1), 26.2 (CH_3_), 52.3 (d, ^2^*J*_PC_ = 6.4, POCH_3_), 52.6 (d, ^2^*J*_PC_ = 6.2, POCH_3_), 76.9 (d, ^2^*J*_CF_ = 1.9, C-2), 81.7 (d, ^2^*J*_PC_ = 7.5, C-3), 82.1 (d, ^2^*J*_CF_ = 6.4, C-4), 82.5 (d, ^2^*J*_CF_ = 17.8, C-5), 84.8 (d, ^1^*J*_CF_ = 172.4, C-6), 112.7 (C(CH_3_)_2_). ^31^P NMR (121 MHz; CDCl_3_): δ = 31.65–32.38 (m, P). ^19^F NMR (282 MHz; CDCl_3_) −229.3 (ddd, ^3^*J*_HF_ = 2.2, ^3^*J*_HF_ = 33.2, ^2^*J*_HF_ = 33.2, ^2^*J*_HF_ = 47.1, 1F). ν_max_ (neat): 2954, 1372, 1209, 1025, 819, 503. HRMS (ES+): Calcd. for C_11_H_20_O_4_FPNa [M+Na]^+^: 321.0879, found: 321.0879.

**D-allo-2,5-Anhydro-1-deoxy-1-(dimethoxyphosphinyl)-6-fluoro-3,4-*O*-isopropylidenehexitol (23b).** The same procedure as described for **23a** was repeated with **22b** (70 mg, 0.23 mmol) to afford **23b** as a colourless oil (60 mg, 83.1%). [α]_D_^20^: −18.78° (*c* 2.45 × 10^−3^, CHCl_3_), ^1^H NMR (300 MHz; CDCl_3_): δ = 4.64 (dd, ^3^*J*_HH_ = 3.9, ^3^*J*_HH_ = 6.6, 1H, H-4), 4.49 (ddd, 1H, ^3^*J*_HH_ = 2.8, ^2^*J*_HH_ = 10.4, ^2^*J*_HF_ = 47.6, H-6), 4.48 (dd, 1H, ^3^*J*_HH_ = 4.3, ^3^*J*_HH_ = 6.6, H-3), 4.45 (ddd, 1H, ^3^*J*_HH_ = 3.5, ^2^*J*_HH_ = 10.8, ^2^*J*_HF_ = 47.0, H-6), 4.23 (dddd, 1H, ^3^*J*_HH_ = 4.2, ^3^*J*_HH_ = 6.7, ^3^*J*_HH_ = 6.7, ^3^*J*_HP_ = 11.1, H-2), 4.09 (ddd, 1H, ^3^*J*_HH_ = 3.4, ^3^*J*_HH_ = 3.4, ^2^*J*_HF_ = 28.3, H-5), 3.71 (d, 3H, ^3^*J*_HH_ = 2.2, POCH_3_), 3.67 (d, 3H, ^3^*J*_HH_ = 2.3, POCH_3_), 2.09 (d, 1H, ^2^*J*_HP_ = 18.4, H-1), 2.13 (dd, 1H, ^3^*J*_HH_ = 0.9, ^2^*J*_HP_ = 18.4, H-1), 1.47 (s, 3H, C(CH_3_)_2_), 1.28 (s, 3H, C(CH_3_)_2_). ^13^C NMR (75 MHz; CDCl_3_): δ = 25.9 (CH_3_), 27.7 (CH_3_), 30.1 (d, ^2^*J*_CP_ = 141.1, C-1), 52.7 (d, ^2^*J*_CP_ = 6.5, OCH_3_), 52.9 (d, ^2^*J*_CP_ = 6.5, OCH_3_), 80.5 (d, ^2^*J*_CF_ = 3.4, C-2), 81.2 (d, ^3^*J*_CF_ = 7.4, C-4), 83.2 (d, ^2^*J*_CF_ = 172.7, C-6), 83.5 (d, ^2^*J*_CF_ = 18.3, C-5), 85.7 (d, ^4^*J*_CF_ = 11.3, C-3), 114.9 (C(CH_3_)_2_). ^31^P NMR (162 MHz; CDCl_3_): δ = 30.33−31.09 (m, P). ^19^F NMR (282 MHz; CDCl_3_): δ = −231.0 (ddd, ^3^*J*_HF_ = 28.8, ^2^*J*_HF_ = 47.3, ^2^*J*_HF_ = 47.3, 1F). ν_max_ (neat): 3464, 2956, 1383, 1214, 1030, 822, 513. HRMS (ES+): Calcd. for C_11_H_20_O_4_FPNa [M+Na]^+^: 321.0879, found: 321.0875.

**Cyclohexylammonium salt 8a.** TMSBr (0.18 ml, 1.36 mmol) was added to a solution of **23a** (94.8 mg, 0.34 mmol) in DCM (5 ml). The progress of reaction was monitored by ^31^P NMR. The disappearing of signal at 31.5–32.1.0 ppm indicating that the reaction was gone completion. A solution of TFA:H_2_O (1:1, 1 ml) was then added to the mixture. After 30 min, water (5 ml) was added, the solvents were removed under reduced pressure. To this residue was added water (5 ml) and the solvent was removed under reduced pressure; this was repeated until all trace of TFA was gone. To this residue 5 ml of water was added and adjusted to pH 11 with cyclohexylamine. After removal of solvent, the residue was dissolved in EtOH and about 0.5 volume of acetone was added. The precipitate was filtered off to afford **8a** as colourless needles (97.6 mg, 87.2%). Mp. 168–170 °C, [α]_D_^20^: +18.67° (*c* 1.50 × 10^−3^, MeOH), ^1^H NMR (400 MHz; MeOH-d_4_): δ = 1.07–1.16 (m, 1H, cyclohexyl), 1.19–1.34 (m, 4H, cyclohexyl), 1.57–1.64 (m, 1H, cyclohexyl), 1.70–1.79 (m, 2H, cyclohexyl), 1.88–1.94 (m, 2H, cyclohexyl), 1.87 (ddd, 1H, ^3^*J*_HH_ = 4.9, ^2^*J*_HH_ = 10.1, ^2^*J*_HP_ = 19.1, H-1), 2.04 (ddd, 1H, ^3^*J*_HH_ = 10.1, ^2^*J*_HH_ = 14.2, ^2^*J*_HP_ = 17.1, H-1), 2.91–2.98 (m, 1H, H-1, cyclohexyl), 3.83 (dddd, 1H, ^3^*J*_HH_ = 2.0, ^3^*J*_HH_ = 4.6, ^3^*J*_HH_ = 8.7, ^3^*J*_HF_ = 25.5, H-5), 4.01 (dd, 1H, ^3^*J*_HH_ = 4.6, ^3^*J*_HH_ = 8.7, H-4), 4.10–4.14 (br, 1H, H-3), 4.17 (dddd, 1H, ^3^*J*_HH_ = 2.9, ^3^*J*_HH_ = 4.5, ^3^*J*_HH_ = 4.5, ^3^*J*_HP_ = 9.7, H-2), 4.34 (ddd, 1H, ^3^*J*_HH_ = 4.7, ^2^*J*_HH_ = 10.3, ^2^*J*_HF_ = 47.7, H-6), 4.56 (ddd, 1H, ^3^*J*_HH_ = 2.1, ^2^*J*_HH_ = 10.3, ^2^*J*_HF_ = 48.4, H-6). ^13^C NMR (100 MHz; MeOH-d_4_): δ = 25.4 (2 × C, cyclohexyl), 25.9 (C, cyclohexyl), 31.2 (d, ^1^*J*_CP_ = 129.5, C-1), 31.9 (2 × C, cyclohexyl), 51.5 (C-1, cyclohexyl), 72.9 (d, ^3^*J*_CF_ = 7.9, C-4), 73.4 (C-3), 79.7 (d, ^2^*J*_CP_ = 2.7, C-2), 81.0 (d, ^2^*J*_CF_ = 17.6, C-5), 84.3 (d, ^1^*J*_CF_ = 170.9, C-6). ^31^P NMR (162 MHz; MeOH-d_4_): δ = 18.7 (ddd, ^3^*J*_HP_ = 2.9, ^2^*J*_HP_ = 18.1, ^2^*J*_HP_ = 18.1 ). ^19^F NMR (376 MHz; MeOH-d_4_): δ = −232.4 (ddd, ^3^*J*_HF_ = 25.5, ^2^*J*_HF_ = 47.4, ^2^*J*_HF_ = 47.4, 1F). ν_max_ (KBr): 3199, 2939, 2720, 2012, 1597, 1429, 1389, 1121, 1018, 925. HRMS (ES+): Calcd. for C_12_H_26_NO_6_FP [M+H]^+^: 330.1482, found: 330.1492.

**Cyclohexylammonium salt 8b.** The same procedure as described for preparation of **8a** was repeated with **23b** (53.1 mg, 0.19 mmol) to afford **8b** as white solid (52.1 mg, 83.3%). Mp. 146–148 °C, [α]_D_^20^: +3.09° (*c* 3.55 × 10^−3^, MeOH), ^1^H NMR (400 MHz; MeOH-d_4_): δ = 1.05–1.32 (m, 5H, cyclohexyl), 1.54–1.74 (m, 5H, 1H-1 overlap with 4H-cyclohexyl), 1.87–1.90 (m, 2H, cyclohexyl), 1.99 (ddd, 1H, ^3^*J*_HH_ = 3.7, ^2^*J*_HH_ = 14.1, ^2^*J*_HP_ = 18.8, H-1), 2.83–2.91 (m, 1H, H-1, cyclohexyl), 3.96 (t, ^3^*J*_HH_ = 6.3, H-3), 3.83 (dddd, 1H, ^3^*J*_HH_ = 3.7, ^3^*J*_HH_ = 3.7, ^3^*J*_HH_ = 3.7, ^3^*J*_HF_ = 25.9, H-5), 3.97 (dd, 1H, ^3^*J*_HH_ = 3.9, ^3^*J*_HH_ = 5.5, H-4), 3.95–4.01 (m, 1H, H-2), 4.31 (ddd, 1H, ^3^*J*_HH_ = 4.1, ^2^*J*_HH_ = 10.2, ^2^*J*_HF_ = 47.4, H-6), 4.36 (ddd, 1H, ^3^*J*_HH_ = 3.2, ^2^*J*_HH_ = 10.2, ^2^*J*_HF_ = 47.9, H-6). ^13^C NMR (100 MHz; MeOH-d_4_): δ = 25.5 (2 × C, clohexyl), 26.1 (C, cyclohexyl), 32.8 (2 × C, clohexyl), 35.9 (d, ^1^*J*_CP_ = 127.0, C-1), 51.4 (C-1, cyclohexyl), 73.1 (d, ^3^*J*_CF_ = 5.1, C-4), 77.4 (C-3), 80.7 (d, ^2^*J*_CP_ = 2.6, C-2), 84.1 (d, ^2^*J*_CF_ = 18.5, C-5), 84.3 (d, ^1^*J*_CF_ = 170.5, C-6). ^31^P NMR (162 MHz; MeOH-d_4_): δ = 17.9 (ddd, ^3^*J*_HP_ = 3.9, ^2^*J*_HP_ = 17.8, ^2^*J*_HP_ = 17.8 ). ^19^F NMR (376 MHz; MeOH-d_4_): δ = −231.6 (ddd, 1F, ^3^*J*_HF_ = 25.6, ^2^*J*_HF_ = 47.7, ^2^*J*_HF_ = 47.7). ν_max_ (KBr): 3436, 2937, 2561, 2012, 1618, 1454, 1389, 1051, 974. HRMS (ES+): Calcd. for C_12_H_26_NO_6_FP [M+H]^+^: 330.1482, found: 330.1485.

### Fluoromethylphostone 10

Deprotection as described for **8a** was repeated with **23a** (80 mg, 0.27 mmol) in DCM (5 ml). Without addition of cyclohexylamine, the residue was dissolved in dry pyridine (1 ml) and acetic anhydride (0.04 ml, 0.4 mmol) was then added. The emergence of an ^31^P-NMR signal at 45 ppm and a disappearance of signal at 27 ppm indicated that the reaction had gone completion. After removal of the solvent under reduced pressure, the crude was purified over silica gel eluting with MeOH:DCM (3:2) to afford **10** as white solid (23 mg, 40.2%). Mp. 180−182 °C, [α]_D_^20^: +103.0° (*c* 1.0 × 10^−3^, MeOH). ^1^H NMR (300 MHz; MeOH-d_4_): δ = 1.73–1.98 (m, 2H, H-1), 4.25 (dddd, 1H, ^3^*J*_HH_ = 2.10, ^3^*J*_HH_ = 3.90, ^3^*J*_HH_ = 8.30, ^3^*J*_HF_ = 26.32, H-1, H-5), 4.36 (ddd, 1H, ^3^*J*_HH_ = 3.90, ^2^*J*_HH_ = 10.63, ^2^*J*_HF_ = 47.18, H-6), 4.48 (ddd, 1H, ^3^*J*_HH_ = 2.10, ^2^*J*_HH_ = 10.63, ^2^*J*_HF_ = 48.14, H-6), 4.67–4.71 (m, 1H, H-3), 4.72–4.82 (m, 1H, H-2), 4.88 (ddd, 1H, ^3^*J*_HH_ = 1.91, ^3^*J*_HH_ = 4.61, ^3^*J*_HP_ = 8.34, H-4). ^13^C NMR (75 MHz; MeOH-d_4_): δ = 29.6 (d, ^1^*J*_CP_ = 119.5, C-1), 74.4 (d, ^4^*J*_CF_ = 7.15, C-4), 77.8 (d, ^4^*J*_CF_ = 8.8, C-3), 79.5 (d, ^3^*J*_CF_ = 18.36, C-5), 81.2 (d, ^2^*J*_CP_ = 4.0, C-2), 83.2 (d, ^1^*J*_CF_ = 172.1, C-6). ^19^F NMR (282 MHz; MeOH-d_4_): δ = −234.3 (ddd, 1F, ^3^*J*_HF_ = 26.4, ^2^*J*_HF_ = 47.4, ^2^*J*_HF_ = 47.7). ^31^P NMR (162 MHz; MeOH-d_4_): δ = 41.9–42.6 (br, m). ν_max_ (PTFE): 3419, 1214, 1154, 799, 503. HRMS (ES-): Calcd. for C_6_H_9_FO_5_P [M-H]: 211.0172, found: 211.0166.

## Supporting Information

The experimental and analytical data for compounds **9b**, **11**, **14**, **16**, and **19**–**22** are provided in the supporting information.

File 1 Experimental.
