# Supplementary material for: Synthesis of phosphonate and phostone analogues of ribose-1-phosphates
Source: Beilstein J Org Chem. 2009 Jul 27;5:37. doi: 10.3762/bjoc.5.37 (PMC2748706; doi:10.3762/bjoc.5.37)
Supplement: File 1 — Experimental. [file Beilstein_J_Org_Chem-05-37-s001.doc]

Supporting Information

for:

Synthesis of phosphonate and phostone analogues of ribose-1-phosphates

Pitak Nasomjai, David O'Hagan* and Alexandra M. Z. Slawin

Address: ^1^School of Chemistry and Centre for Biomolecular Sciences, University of St Andrews, North Haugh, St Andrews, Fife, KY16 9ST, UK.

Email: David O'Hagan* - do1@st-andrews.ac.uk

* Corresponding author

**Experimental**

**General**

Nuclear magnetic resonance (NMR) spectra were obtained using Bruker Advance 300 and Bruker Advance II 400. All chemical shifts (δ) are reported in parts per million (ppm) and coupling constants (*J*) are given in Hertz (Hz). Melting points were determined in Pyrex capillaries using a Gallenkamp Griffin MPA350.BM2.5 melting point apparatus. Infra red (IR) spectra were recorded on Nicolet Avatar 360 FT-IR. High-mass resolution spectrometry (HRMS) was carried out using a Micromass LCT mass spectrometer with electrospray ionization (ESI) operating in positive and negative modes. Optical rotations were measured on Perkin-Elmer model 341 polarimeter.

**2,3-*O*-Isopropylidene-D-ribono-1,4-lactone (14)** [1]

Catalytic amount of concentrated sulfuric acid was added to a suspension of D-ribono-γ-1,4-lactone **12** (2.92 g, 19.75 mmol) in acetone (100 cm^3^) at 0 ˚C. After warmed to ambient temperature and stirred for further 4 h, 35%NH_3_ was slowly added until the solution neutralised, the precipitate was then filtered off. Recrystallisation of the solid from benzene yielded **14** as colourless needle (3.19 g, 86%); mp: 136–137 °C. (lit. [1] 138–139 °C), [α]^20^_D_ : −62.3 (*c* 0.05, acetone) (lit. [1] [α]^20^_D_ −84.2° (*c* 0.9, acetone); ^1^H NMR (400 MHz; Acetone-d_6_): δ = 1.35 (s, 3H, CH_3_), 1.39 (s, 3H, CH_3_), 3.83 (m, 2H, H-5), 4.59 (t, 1H, ^3^*J*_HH_ = 2.3, H -4), 4.77 (d, 1H, ^3^*J*_HH_ = 5.6, H-3), 4.85 (d, 1H, ^3^*J*_HH_ = 5.6, H-2). ^13^C NMR (100 MHz; acetone-d_6_): δ = 25.5 (CH_3_), 27.1 (CH_3_), 62.1 (C-5), 79.3 (C-4), 83.1 (C-2), 76.4 (C-3), 112.9 (C(CH_3_)_2_), 206.3 (C-1). ν_max_ 3460, 1771, 1201, 1088, 968 cm^−1^. *m/z* (ES+) 210.99 [M+Na] (100%).

**(2*S*,3*R*,4*S*)-1-Fluoro-2-hydroxy-3,4-(*O*-isopropylidenedioxy)-pentan-5-ol (16)**

NaBH_4_ (11.18 mg, 0.3 mmol) was added to a vigorously stirred solution of **15** (22.5 mg, 0.12 mmol) in absolute ethanol (3 cm^3^) at 0 °C. After stirring for 2 h, excess AcOH was added, and the volatiles were evaporated under reduced pressure. The residue was dissolved in EtOAc (20 cm^3^), the solution was washed with H_2_O, dried over Mg_2_SO_4_, After removal of solvent, the residue was purified over silica gel eluting with EtOAc:cyclohexane (1:4) to afford **16** as colourless oil (14.97 mg, 65.3%). [α]^20^_D_ +19.6˚ (*c* 4.55 × 10^−3^, CHCl_3_). ^1^H NMR (400 MHz; CDCl_3_): δ = 1.35 (s, 3H, CH_3_), 1.41 (s, 3H, CH_3_), 3.73 (dd, 1H, *^3^J*_HH_ = 4.7, ^2^*J*_HH_ = 11.6, H-1), 3.81 (dd, 1H, ^3^*J*_HH_ = 3.8, ^2^*J*_HH_ = 11.6, H-1), 3.96 (dddd, ^3^*J*_HH_ = 2.3, ^3^*J*_HH_ = 5.5, ^3^*J*_HH_ = 9.7, ^3^*J*_HF_ = 21.7, H-4), 4.07 (dd, ^3^*J*_HH_ = 5.8, ^3^*J*_HH_ = 9.7, H-3), 4.29 (dddd, 1H, ^3^*J*_HH_ = 5.3, ^3^*J*_HH_ = 5.3, ^3^*J*_HH_ = 7.6, ^3^*J*_HH_ = 9.7, H-2), 4.48 (ddd, 1H, ^3^*J*_HH_ = 5.5, ^2^*J*_HH_ = 9.7, ^2^*J*_HF_ = 47.5, H-5), 4.60 (ddd, 1H, ^3^*J*_HH_ = 2.3, ^2^*J*_HH_ = 9.7, ^2^*J*_HF_ = 49.8, H-5). ^13^C (100 MHz; CDCl_3_): δ = 25.5 (CH_3_), 26.8 (CH_3_), 61.1 (C-1), 69.2 (d, ^2^*J*_CF_ = 17.7, C-4), 75.8 (d, ^3^*J*_CF_ = 6.7, C-3), 77.5 (C-2), 85.5 (d, ^1^*J*_CF_ = 163.8, C-5), 109.5 (C(CH_3_)_2_).^19^F (282 MHz; CDCl_3_): δ = −236.5 (ddd, ^3^*J*_HF_ = 22.1, ^2^*J*_HF_ = 47.4, ^2^*J*_HF_ = 47.4). ν_max_ (neat): 3384, 2988, 1373, 1043, 874, 507. HRMS (ES+): Calcd. for C_8_H_15_FO_4_Na^+^ [M+Na]^+^: 217.0852, found: 217.0848.

**2,3-*O*-Isopropylidene-D-ribofuranose (19)** [2]

Catalytic amount of concentrated sulfuric acid was added to a suspension of D-ribose **13** (3 g, 19.98 mmol) in acetone (100 cm^3^) at 0 °C. After warmed to ambient temperature and stirred for further 4 h, solid NaHCO_3_ was slowly added until the solution neutralised. Filtration and evaporation of solvents under reduced pressure yielded the crude product. The purification of the crude product over silica gel eluting with 60% EtOAc:hexane gave **19** as colourless syrup (3.43 g, 90.1%); [α]^20^_D_ : −62.3° (*c* 0.05, acetone) (lit. [2] [α]^20^_D_ −36.2 (*c* 1.45, acetone); ^1^H NMR (300 MHz; MeOH-d_4_): δ = 1.31 (3 H, s, CH_3_), 1.44 (3 H, s, CH_3_), 3.58 (dd, 1 H, *^3^J*_HH_ = 5.38, ^2^*J*_HH_ = 11.68, H-5), 3.63 (dd, 1 H, *^3^J*_HH_ = 4.71, ^2^*J*_HH_ = 11.68, H-5), 4.19 (t, 1 H, ^3^*J*_HH_ = 4.90, H-4), 4.52 (d, 1 H, ^3^*J*_HH_ = 5.69, H-2), 4.77 (d, 1 H, ^3^*J*_HH_ = 5.93, H-3), 5.26 (br, s, 1H, H-1); ^13^C NMR (75 MHz; MeOh-d_4_): δ = 25.4 (CH_3_), 27.2 (CH_3_), 64.7 (C-5), 83.8 (C-3), 88.3 (C-2), 88.9 (C-4), 104.3 (C-1), 113.6 (C(CH_3_)_2_). ν_max_ (PTFE): 3445, 2986, 1372, 1212 *m/z* (ES+) 211.28 [M+Na] (100%), 149.20 [M-C(CH_3_)_2_] (40%).

**2,3-*O*-Isopropylidene-5-*O*-trityl-D-ribofuranose (20)** [3]

Trityl chloride (3.67 g, 13.14 mmol) was added portionwise to a solution of **19** (2.50 g, 13.14 mmol) in triethylamine (3.67 ml, 26.28 mmol). Catalytic amount of DMAP was then added. After stirring overnight, water (20 ml) was added and the mixture was extracted with EtOAc (3 × 20 ml). The combined organic phases were washed with water, brine, dried over MgSO_4_, and filtered. After removal of solvent, the residue was purified over silica gel eluting with EtOAc:hexane (1:4) to afford **20** as viscous oil (5.30g, 93.6%, α/β = 1/4.3). **α-anomer**: ^1^H NMR (300 MHz; CDCl_3_): δ = 1.29 (s, CH_3_),1.48 (s, CH_3_), 2.94 (dd, ^3^*J*_HH_ = 2.8, ^3^*J*_HH_*­* = 10.2, H-5), 3.38 (dd, ^3^*J­*_HH_ = 3.1, ^3^*J­*_HH_ = 10.2, H-5), 3.93 (d, ^3^*J*_HH_ = 11.3, -OH), 4.12 (t, ^3^*J­*_HH_ = 2.6, H-4), 4.51 (dd, ^3^*J*_HH_*­* = 0.9, ^3^*J­*_HH_ = 6.3, H-3), 4.67 (dd, ^3^*J*_HH_*­* = 4.1, ^3^*J*_HH_*­* = 6.3, H-2), 5.67 (dd, ^3^*J­*_HH_ = 4.1, ^3^*J­*_HH_ = 11.3, H-1), 7.17–7.35 (m, 15H, Ar-H, overlap with β-anomer). ^13^C (75 MHz; CDCl_3_): δ = 25.2 (CH_3_), 26.6 (CH_3_), 65.8 (C-5), 79.9 (C-3), 82.6 (C-4), 86.5 (C-2), 98.5 (C-1), 113.5 (C(CH_3_)_2_), 127.6 (Ar-C), 128.4 (Ar-C), 129.0 (Ar-C), 143.9 (Ar-C_quat_). **β-anomer**: ^1^H NMR (300 MHz; CDCl_3_): δ = 1.28 (s, CH_3_), 1.41 (s, CH_3_), 3.27 (dd, ^3^*J*_HH_ = 3.8, ^2^*J*_HH_ = 10.4, H-5), 3.35 (dd, ^3^*J*_HH_ = 3.5, ^2^*J*_HH_ = 10.4, H-5), 3.86 (d, ^3^*J*_HH_ = 9.1, -OH), 4.28 (t, ^3^*J*_HH_ = 3.5, H-4), 4.59 (d, ^3^*J*_HH_ = 5.9, H-3), 4.71 (dd, ^3^*J*_HH_ = 0.6, ^3^*J*_HH_ = 5.9, H-2), 5.26 (d, ^3^*J*_HH_ = 9.1, H-1), 7.17–7.35 (m, 15H, Ar-H, overlap with α-anomer). ^13^C NMR (75 MHz; CDCl_3_): δ = 25.5 (Me), 26.9 (Me), 65.5 (C-5), 80.5 (C-3), 82.4 (C-4), 87.5 (C-2), 103.9 (C-1), 112.7 (C(CH_3_)_2_), 127.9 (Ar-C), 128.5 (Ar-C), 129.1 (Ar-C), 143.2 (Ar-C_quat_). ν_max_(neat): 3439, 3058, 2339, 1569, 1491, 1448, 1374, 1212, 1072, 870, 705, 648. *m/z* (ES+) 455.06 [M+Na] (40%); 243.03 [Ph_3_C] (100%).

**D-altro-2,5-Anhydro-1-deoxy-1-(dimethoxyphosphinyl)-3,4-*O*-isopropylidene-6-*O*-tritylhexitol (21a) and D-allo-2,5-Anhydro-1-deoxy-1-(dimethoxyphosphinyl)-3,4-*O*-isopropylidene-6-*O*-tritylhexitol (21b)** [3]

The same procedure as described for preparation of **18** was repeated with **20** (2.1g, 5 mmol) to afford **21a** (1.36g, 50.64%) as viscous colourless oil and **21b** as viscous colourless oil (0.32 g, 13.39%). **21a**: [α]_D_^20^: +2.9° (*c* 11.45 × 10^−3^, CHCl_3_), ^1^H NMR (300 MHz; CDCl_3_): δ = 1.35 (s, 3H, CH_3_), 1.51 (s, 3H, CH_3_), 2.26 (ddd, 1H, ^3^*J*_HH_ = 7.0, ^2^*J*_HH_ = 15.5, ^2^*J*_HP_ = 17.6, H-1), 2.36 (ddd, 1H, ^3^*J*_HH_ = 6.2, ^2^*J*_HH_ = 15.5, ^2^*J*_HP_ = 18.5, H-1), 3.15 (dd, 1H, ^3^*J*_HH_ = 4.8, ^2^*J*_HH_ = 10.1, H-6), 3.28 (dd, 1H, ^3^*J*_HH_ = 4.2, ^2^*J*_HH_ = 10.1, H-6), 3.75 (d, 3H, ^3^*J*_HP_ = 3.8, POCH_3_), 3.79 (d, 3H, ^3^*J*_HP_ = 3.8, POCH_3_), 4.23 (t, 1H, ^3^*J*_HH_ = 4.4, H-5), 4.57 (dddd, 1H, ^3^*J*_HH_ = 3.8, ^3^*J*_HH_ = 6.4, ^3^*J*_HH_ = 6.4, ^3^*J*_HP_ = 10.1, H-2), 4.69 (d, ^3^*J*_HH_ = 6.0, 1H, H-4), 4.76 (dd, ^3^*J*_HH_ = 3.9, ^3^*J*_HH_ = 6.0, 1H, H-3), 7.23–7.28 (m, 3H, Ar-H), 7.30–7.36 (m, 6H, Ar-H), 7.41–7.46 (m, 6H, Ar-H). ^31^C NMR (75 MHz; CDCl_3_): δ = 25.6 (CH_3_), 26.1 (d, ^1^*J*_PC_ = 141.2, C-1), 27.0 (CH_3_), 52.6 (d, ^2^*J*_CP_ = 6.3, OCH_3_), 53.2 (d, ^2^*J*_CP_ = 6.3, OCH_3_), 64.8 (C-6) 77.1 (C-3), 82.4 (d, ^2^*J*_CP_ = 8.3, C-2), 83.7 (C-4), 83.8 (C-5), 112.8 (C(CH_3_)_2_), 127.5 (Ar-C), 128.3 (Ar-C), 129.0 (Ar-C), 143.9 (Ar-C). ^31^P NMR (121 MHz; CDCl_3_): δ = 32.3–33.0 (m, P). ν_max_(neat): 2949, 1489, 1447, 1371, 1209, 1029, 705, 503. HRMS (ES+): Calcd. for C_30_H_35_O_7_PNa [M+Na]^+^: 561.2018, found: 561.2021. **10b**: [α]_D_^20^: −8.3° (*c* 12.85 × 10^−3^, CHCl_3_), ^1^H NMR (300 MHz; CDCl_3_): δ = 1.35 (s, 3H, CH_3_), 1.55 (s, 3H, CH_3_), 2.16 (ddd, 1H, ^3^*J*_HH_ = 7.7, ^2^*J*_HH_ = 15.3, ^2^*J*_HP_ = 17.8, H-1), 2.24 (ddd, 1H, ^3^*J*_HH_ = 5.6, ^2^*J*_HH_ = 15.3, ^2^*J*_HP_ = 19.1, H-1), 3.19 (dd, 1H, ^3^*J*_HH_ = 4.8, ^2^*J*_HH_ = 11.1, H-6), 3.33 (dd, 1H, ^3^*J*_HH_ = 3.7, ^2^*J*_HH_ = 11.0, H-6), 3.74 (d, 3H, ^3^*J*_HP_ = 11.0, POCH_3_), 3.78 (d, 3H, ^3^*J*_HP_ = 11.0, POCH_3_), 4.20 (dd, 1H, ^3^*J*_HH_ = 3.4, ^3^*J*_HH_ = 8.3, H-5), 4.26 (dddd, 1H, ^3^*J*_HH_ = 5.3, ^3^*J*_HH_ = 5.3, ^3^*J*_HH_ = 7.7, ^3^*J*_HP_ = 10.3, H-2), 4.54 (dd, 1H, ^3^*J*_HH_ = 5.1, ^3^*J*_HH_ = 6.6, H-3), 4.62 (dd, ^3^*J*_HH_ = 3.5, ^3^*J*_HH_ = 6.6, 1H, H-4), 7.22–7.28 (m, 3H, Ar-H), 7.28–7.35 (m, 6H, Ar-H), 7.44–7.48 (m, 6H, Ar-H). ^13^C NMR (75 MHz; CDCl_3_): δ = 26.1 (CH_3_), 27.1 (CH_3_), 30.4 (d, ^1^*J*_CP_ = 141.1, C-1), 52.7 (d, ^2^*J*_CP_ = 6.4, OCH_3_), 53.1 (d, ^2^*J*_CP_ = 6.4, OCH_3_), 64.7 (C-6), 79.9 (d, ^2^*J*_CP_ = 3.6, C-3), 82.7 (C-4), 83.9 (C-5), 85.7 (d, ^2^*J*_CP_ = 12.6, C-2), 114.9 (C(CH_3_)_2_), 127.5 (Ar-C), 128.3 (Ar-C), 129.1 (Ar-C), 144.1 (Ar-C). ^31^P NMR (121 MHz; CDCl_3_): δ = 30.6-31.7 (m, P). ν_max_(neat): 2955, 1486, 1442, 1374, 1211, 1032, 711, 513. HRMS (ES+): Calcd. for C_30_H_35_O_7_PNa [M+Na]^+^: 561.2018, found: 561.2018.

**D-altro-2,5-Anhydro-1-deoxy-1-(dimethoxyphosphinyl)-3,4-*O*-isopropylidenehexitol (22a)**

Anhydrous ZnCl_2_ (3.1g, 22.6 mmol) was added portionwise to a solution of **21a** (1.22 g, 2.26 mmol) in DCM (40 ml) over 1 h. The reaction was monitored by TLC showing that starting material was consumed. After completion, sat. NH_4_Cl (10 ml) was added and the reaction mixture was extracted into DCM (3 × 10 ml), the combined organic phases were washed with water, brine, and died over MgSO_4_. After removal of solvent, the residue was purified over silica gel eluting with MeOH:DCM (1:20) to afford **22a** as viscous oil (0.53 g, 78.2%). [α]_D_^20^: +1.7° (*c* 10.85 × 10^−3^, CHCl_3_), ^1^H NMR (400 MHz; CDCl_3_): δ = 1.27 (s, 3H, CH_3_), 1.42 (s, 3H, CH_3_), 2.14 (ddd, 1H, ^3^*J*_HH_ = 7.3, ^2^*J*_HH_ = 15.5, ^2^*J*_HP_ = 17.7, H-1), 2.18 (ddd, 1H, ^3^*J*_HH_ = 6.2, ^2^*J*_HH_ = 15.3, ^2^*J*_HP_ = 18.5, H-1), 3.56 (d, 2H, ^3^*J*_HH_ = 5.5, H-6), 3.68 (d, 3H, ^3^*J*_HP_ = 10.9, POCH_3_), 3.70 (d, 3H, ^3^*J*_HP_ = 10.9, POCH_3_), 4.06 (t, 1H, ^3^*J*_HH_ = 5.2, H-5), 4.25 (dddd, 1H, ^3^*J*_HH_ = 3.8, ^3^*J*_HH_ = 6.5, ^3^*J*_HH_ = 6.5, ^3^*J*_HP_ = 10.1, H-2), 4.61 (dd, ^3^*J*_HH_ = 3.7, ^3^*J*_HH_ = 6.2, 1H, H-3), 4.65 (d, ^3^*J*_HH_ = 6.2, 1H, H-4). ^13^C NMR (100 MHz; CDCl_3_): δ = 25.0 (CH_3_), 26.3 (d, ^1^*J*_CP_ = 141.8, C-1), 26.4 (CH_3_), 52.2 (d, ^2^*J*_PC_ = 6.5, OCH_3_), 52.9 (d, ^2^*J*_PC_ = 6.1, OCH_3_), 62.1 (C-6), 75.8 (C-2), 81.8 (d, ^2^*J*_PC_ = 9.3, C-3), 82.7 (C-4), 84.7 (C-5), 112.5 (C(CH_3_)_2_). ^31^P NMR (162 MHz; CDCl_3_): δ = 31.5-32.1.0 (m, P). ν_max_(neat): 3385, 2954, 1373, 1212, 1032, 826, 537. HRMS (ES+): Calcd. for C_11_H_21_O_7_PNa [M+Na]^+^: 319.0923, found: 319.0927.

**D-allo-2,5-Anhydro-1-deoxy-1-(dimethoxyphosphinyl)-3,4-*O*-isopropylidenehexitol (22b)**

The same procedure as described for **22a** was repeated with **21b** (0.29 g, 0.54 mmol) to afford **22b** as viscous colourless oil (0.13 g, 80.6 %). [α]_D_^20^: −21.5° (*c* 9.75 × 10^−3^, CHCl_3_), ^1^H NMR (400 MHz; CDCl_3_): δ = 1.27 (s, 3H, CH_3_), 1.46 (s, 3H, CH_3_), 2.12 (ddd, 1H, ^3^*J*_HH_ = 6.4, ^2^*J*_HH_ = 15.5, ^2^*J*_HP_ = 18.1, H-1), 2.19 (ddd, 1H, ^3^*J*_HH_ = 5.6, ^2^*J*_HH_ = 15.5, ^2^*J*_HP_ = 18.9, H-1), 3.56 (dd, 1H, ^3^*J*_HH_ = 2.8, ^2^*J*_HH_ = 12.5, H-6), 3.67 (d, 3H, ^3^*J*_HP_ = 11.0, POCH_3_), 3.71 (d, 3H, ^3^*J*_HP_ = 11.0, POCH_3_), 3.72 (dd, 1H, ^3^*J*_HH_ = 2.9, ^2^*J*_HH_ = 12.2, H-6), 4.08 (dd, 1H, ^3^*J*_HH_ = 3.1, ^3^*J*_HH_ = 6.2, H-5), 4.17 (dddd, 1H, ^3^*J*_HH_ = 5.7, ^3^*J*_HH_ = 5.7, ^3^*J*_HH_ = 5.7, ^3^*J*_HP_ = 19.3, H-2), 4.53 (dd, 1H, ^3^*J*_HH_ = 4.7, ^3^*J*_HH_ = 6.4, H-3), 4.71 (dd, ^3^*J*_HH_ = 2.9, ^3^*J*_HH_ = 6.4, 1H, H-4). ^13^C NMR (100 MHz; CDCl_3_): δ = 25.4 (CH_3_), 27.3 (CH_3_), 29.1 (d, ^2^*J*_PC_ = 141.9, C-1), 52.1 (d, ^2^*J*_PC_ = 6.3, POCH_3_), 52.9 (d, ^2^*J*_PC_ = 6.3, POCH_3_), 62.7 (C-6), 79.3 (d, ^2^*J*_PC_ = 6.5, C-2), 81.7 (C-4), 84.9 ( d, ^3^*J*_PC_ = 10.4, C-3), 85.4 (C-5), 113.8 (CCH_3_)_2_). ^31^P NMR (162 MHz; CDCl_3_): δ = 30.80–31.43 (m, P).**ν_max_**: 3386, 2936, 1381, 1031, 826. HRMS (ES+): Calcd. for C_11_H_21_O_7_PNa [M+Na]^+^: 319.0923, found: 319.0925.

**Cyclohexylammonium salt 14a** [3,4]

TMSBr (0.08 ml, 0.64 mmol) was added to a solution of **22a** (44.8 mg, 0.16 mmol) in DCM (5 ml). The progress of reaction was monitored by ^31^P NMR. The disappearing of signal at 31.5–32.1.0 ppm indicating that the reaction was gone completion. A solution of TFA:H_2_O (1:1, 1 ml) was then added to the mixture. After 30 min, water (5 ml) was added, the solvent were removed under reduced pressure. To this residue was added water (5 ml) and the solvent was removed under reduced pressure; this was repeated until all trace of TFA was gone. To this residue 5 ml of water was added and adjusted pH to 11 with cyclohexylamine. After removal of solvent, the residue was dissolved in EtOH and about 0.5 volume of acetone was added. The precipitate was filtered off to afford **9a** as white solid (43.8 mg, 83.7%). [α]_D_^20^: +1.0° (*c* 8.45 × 10^−3^, MeOH), ^1^H NMR (400 MHz; MeOH-d_4_): δ = 1.18–1.27 (m, 1H, cyclohexyl). 1.31–1.44 (m, 4H, cyclohexyl), 1.71 (br d, 1H, ^2^*J*_HH_ = 12.6, cyclohexyl), 1.80–2.07 (m, 6H, 2H-1 overlap with 4H-cyclohexyl), 3.06 (br, s, 1H, cyclohexyl), 3.54 (dd, 1H, ^3^*J*_HH_ = 5.0, ^2^*J*_HH_ = 11.9, H-5), 3.68 (dd, 1H, ^3^*J*_HH_ = 3.5, ^2^*J*_HH_ = 11.9, H-5), 3.78–3.82 (m, 2H, H-3 and H-5), 4.01 (t, ^3^*J*_HH_ = 5.0, 1H, H-4), 4.06 (quintet, 1H, ^3^*J*_HH_ = 6.9, H-2). ^13^C NMR (75 MHz; MeOH-d_4_): δ = 25.7 (2 × C), 25.4 (C), 32.4 (2 × C), 51.8 (C-1cyclohexyl), 35.4 (d, ^1^*J*_CP_ = 130.8, C-1), 64.0 (C-6), 73.5 (C-4), 77.9 (d, ^2^*J*_CP_ = 6.5, C-2), 80.7 (C-3), 86.3 (C-5). ^31^P NMR (162 MHz; MeOH-d_4_): δ = 19.4 (ddd, ^3^*J*_HP_ = 6.9, ^2^*J*_HP_ = 17.5, ^2^*J*_HP_ = 17.5). ν_max_(KBr): 2938, 2013, 1638, 1389, 1123, 1050, 906. HRMS (ES+): Calcd. for C_12_H_27_NO_7_P [M+H]^+^: 328.1525, found: 328.1522.

**Cyclohexylammonium salt 9b** [3,4]

The same procedure as described for preparation of **9a** was repeated with **22b** (83.2 mg, 0.3 mmol) to afford **9b** (84.1 mg, 85.6%). [α]_D_^20^: +1.6° (*c* 5.65 × 10^−3^, MeOH), ^1^H NMR (400 MHz; MeOH-d_4_): δ = 1.07–1.15 (m, 1H, cyclohexyl), 1.21–1.32 (m, 4H, cyclohexyl), 1.59 (br d, 1H, ^2^*J*_HH_ = 12.5, cyclohexyl), 1.68–1.98 (m, 6H, 2H-1 overlap with 4H-cyclohexyl), 2.95 (br s, 1H, cyclohexyl), 3.45 (dd, 1H, ^3^*J*_HH_ = 5.3, ^2^*J*_HH_ = 11.9, H-5), 3.59 (dd, 1H, ^3^*J*_HH_ = 3.5, ^2^*J*_HH_ = 11.9, H-5), 3.67–3.72 (m, 2H, H-4 and H-5), 3.09 (t, ^3^*J*_HH_ = 4.9, 1H, H-3), 3.96 (quintet, 1H, ^3^*J*_HH_ = 6.8, H-2). ^13^C NMR (75 MHz; MeOH-d_4_): δ = 25.5 (2 × C), 25.9 (1C), 32.0 (2 × C), 35.1 (d, ^1^*J*_CP_ = 129.9, C-1), 51.3 (C-1-cyclohex), 63.7 (C-6), 73.0 (C-4), 77.6 (d, ^2^*J*_CP_ = 7.1, C-2), 80.4 (C-3), 85.6 (C-5). ^31^P NMR (162 MHz; MeOH-d_4_): δ = 19.6-19.8 (br, m). ν_max_(KBr): 3200, 2938, 2060, 2021, 1596, 1492, 1389, 1018. HRMS (ES+): Calcd. for C_12_H_27_NO_7_P [M+H]^+^: 328.1525, found: 328.1515.

**Phostone 11**

Deprotection as described for **9a** was repeated with **22a** (50 mg, 0.17 mmol) in DCM (5 ml). Without basification, the residue was dissolved in dry pyridine (1 ml) and acetic anhydride (0.027 ml, 0.28 mmol) was then added. An emergence of signal at 45 ppm and a disappearance of signal at 27 ppm indicating that the reaction was gone completion. After removal of solvent under reduced pressure, the crude was purified over silica gel eluting with MeOH:DCM (3:2) to afford **11** as white solid (16 mg, 47.1%). Mp. 168–170 ˚C, [α]_D_^20^: +10.5˚ (*c* 2.05 × 10^−3^, MeOH), ^1^H NMR (300 MHz; MeOH-d_4_): δ = 1.84 (ddd, 1H, ^3^*J*_HH_ = 2.1, ^2^*J*_HP_ = 12.5, ^2^*J*_HH_ = 15.2, H-1), 1.98 (ddd, 1H, ^3^*J*_HH_ = 6.9, ^2^*J*_HP_ = 13.7, ^2^*J*_HH_ = 15.2, H-1), 3.58 (dd, 1H, ^3^*J*_HH_ = 4.5, ^2^*J*_HH_ = 12.1, H-6), 3.75 (dd, 1H, ^3^*J*_HH_ = 2.3, ^2^*J*_HH_ = 12.1, H-6), 3.95 (ddd, 1H, ^3^*J*_HH_ = 2.5, ^3^*J*_HH_ = 4.4, ^3^*J*_HP_ = 8.6, H-5), 4.05 (ddd, 1H, ^3^*J*_HH_ = 1.8, ^3^*J*_HH_ = 4.4, ^3^*J*_HP_ = 8.5, H-4), 4.51 (ddd, 1H, ^3^*J*_HH_ = 2.8, ^3^*J*_HH_ = 4.2, ^3^*J*_HH_ = 4.2, H-3), 4.77 (dddd, 1H, ^3^*J*_HH_ = 2.1, ^3^*J*_HH_ = 4.1, ^3^*J*_HH_ = 6.4, ^3^*J*_HP_ = 22.9, H-2). ^13^C NMR (75 MHz; MeOH-d_4_): δ = 30.5 (d, ^1^*J*_CP_ = 119.1, C-1), 62.9 (C-6), 74.1 (d, ^4^*J*_CP_ = 6.9, C-4), 80.6 (d, ^3^*J*_CP_ = 3.3, C-3), 80.7 (d, ^2^*J*_CP_ = 14.9, C-2), 83.2 (C-5). ^31^P NMR (162 MHz; MeOH-d_4_): δ = 43.1–44.5 (br, m). ν_max_ (KBr): 3570, 1736, 1215, 1154, 503. HRMS (ES-): Calcd. for C_6_H_10_O_6_P [M-H]: 209.0215, found: 209.0211.

## References

1. Kandil, A. A.; Slessor, K. N. *J. Org. Chem.* **1985,** *50,* 5649–5655. doi:10.1021/jo00350a045
2. Moon, H. R.; Choi, W. J.; Kim, H. O.; Jeong, L. S. *Tetrahedron: Asymmetry* **2002,** *13,* 1189–1193. doi:10.1016/S0957-4166(02)00316-6
3. Meyer, R. L.; Stone, T. E.; Jesthi, P. K. *J. Med. Chem.* **1984,** *27,* 1095–1098. doi:10.1021/jm00374a028
4. McClard, R. W.; Witte, J. F. *Bioorg. Med. Chem. Lett.* **1994,** *4,* 1537–1538.

doi:10.1016/S0960-894X(01)80528-X
